# YAP1 contributes to NSCLC invasion and migration by promoting Slug transcription via the transcription co-factor TEAD

**DOI:** 10.1038/s41419-018-0515-z

**Published:** 2018-04-27

**Authors:** Mengxue Yu, Yingzhun Chen, Xuelian Li, Rui Yang, Lijia Zhang, Longtao Huangfu, Nan Zheng, Xiaoguang Zhao, Lifang Lv, Yaozhen Hong, Haihai Liang, Hongli Shan

**Affiliations:** 1 Department of Pharmacology (State-Province Key Laboratories of Biomedicine-Pharmaceutics of China, Key Laboratory of Cardiovascular Research, Ministry of Education), College of Pharmacy, Harbin Medical University, 150081 Harbin, Heilongjiang P. R. China; 2 Department of Pathology, the 2nd Affiliated Hospital, Harbin Medical University, Harbin, Heilongjiang P. R. China; 3 Northern Translational Medicine Research and Cooperation Center, Heilongjiang Academy of Medical Sciences, Harbin Medical University, 150081 Harbin, Heilongjiang P. R. China

## Abstract

Yes-associated protein 1 (YAP1) contributes to the development of multiple tumors, but the mechanism underlying YAP1 deregulation in non-small cell lung cancer (NSCLC) remains unclear. By performing immunohistochemistry (IHC) assays, we found that YAP1 was significantly upregulated in NSCLC compared with adjacent tissues; therefore, we sought to elucidate whether the upregulation of YAP1 contributes to NSCLC progression. MTT and transwell assays showed that YAP1 overexpression promoted proliferation, migration, and invasion in the NSCLC cell lines A549 and H460; YAP1 overexpression also promoted the significant differential expression of epithelial-mesenchymal transition (EMT)-related markers. Nevertheless, YAP1 knockdown alleviated TGF-β1-induced EMT and proliferation, migration, and invasion in NSCLC. Furthermore, western blotting showed that the co-transcription complex YAP1/TEAD was impaired by YAPS94A (a YAP1 mutant without the TEAD binding site), and verteporfin (a small molecular inhibitor of YAP1) inhibited A549 and H460 cell metastasis and EMT-related markers expression, indicating that TEAD mediated the NSCLC aggressiveness induced by YAP1. Moreover, sequence analysis and ChIP and luciferase assays confirmed that YAP1 transcriptionally activated Slug expression by binding to TEAD. Importantly, silencing YAP1 inhibited A549 cell tumorigenesis and EMT and downregulated Slug expression in vivo. Overall, our findings revealed that YAP1 is a driver of NSCLC metastasis because YAP1 promoted the EMT program by inducing Slug transcription.

## Introduction

Lung cancer is the leading cause of cancer-associated death around the world^1^, and approximately 80% of cases are histopathologically classified as non-small cell lung cancer (NSCLC)^2^. Due to the early metastasis of NSCLC, the five-year survival rate of patients is lower than 15%. Although there has been progress in uncovering the mechanisms of lung tumorigenesis, our understanding of the molecular mechanisms of NSCLC metastasis remains limited, especially the origin of metastatic traits.

Epithelial mesenchymal transition (EMT), an important cellular development process, is evoked during tumor invasion and metastasis; this process allows the epithelial cells to convert into mesenchymal cells^3,4^. In addition, the inactivation of E-cadherin is considered to be a hallmark of EMT^3,5,6^. The transforming growth factor beta (TGF-β) signaling pathway has been shown to be a major inducer of EMT, thus promoting breast cancer metastasis^7,8^. In addition to TGF-β, several other tyrosine kinase receptors, including insulin-like growth factor (IGF) and platelet-derived growth factor (PDGF), also play critical roles in regulating EMT during tumor progression^9,10^. EMT inducers converge to activate one or more transcription factors (TFs). Those TFs, including SNAI1 and Slug, ZEB1 and ZEB2, and TWIST1 and TWIST2, directly or indirectly suppress the E-cadherin promoter^11–13^.

Hippo signaling is a tumor suppressor pathway that can control organ size and tissue stem cell maintenance^14–17^. Yes-associated protein 1 (YAP1), the key effector of the Hippo pathway, is a highly conserved component of the Hippo pathway in mammalian systems^14^. When YAP1 is active, it localizes to the nucleus and binds to TFs, such as TEAD^18,19^, and drives tumor growth, metastasis, and senescence in cancer cell lines^20–22^. When Hippo signaling is activated, YAP1 is restricted by a kinase cascade, phosphorylated and then degraded in the cytoplasm^23–28^. It has been revealed that YAP1 is involved in the progression of many types of tumors; in fact, YAP1 activation has been established as an independent predictor of hepatocellular carcinoma patient survival^29^, and YAP1 promotes metastasis in gastric cancer^30^. Moreover, YAP1 can also confer cancer stem cell properties by upregulating SOX9 and can inhibit skeletal development and bone repair by affecting chondrocyte proliferation^31,32^. Due to these pleiotropic effects, YAP1 is considered as an essential target of NSCLC, but the molecular mechanisms of YAP1 in NSCLC remain to be elucidated. Furthermore, whether the deregulation of YAP1 contributes to EMT and promotes NSCLC metastasis remains unclear.

Here, we investigated the expression and the mechanistic links that could explain the extraordinary potency of YAP1 in driving tumor metastasis, and we show a direct effect of YAP1 on Slug transcription. Thus, our findings provide new insights into the mechanism of YAP1-induced EMT in NSCLC.

## Results

### YAP1 upregulation in NSCLC

**Fig. 1:**
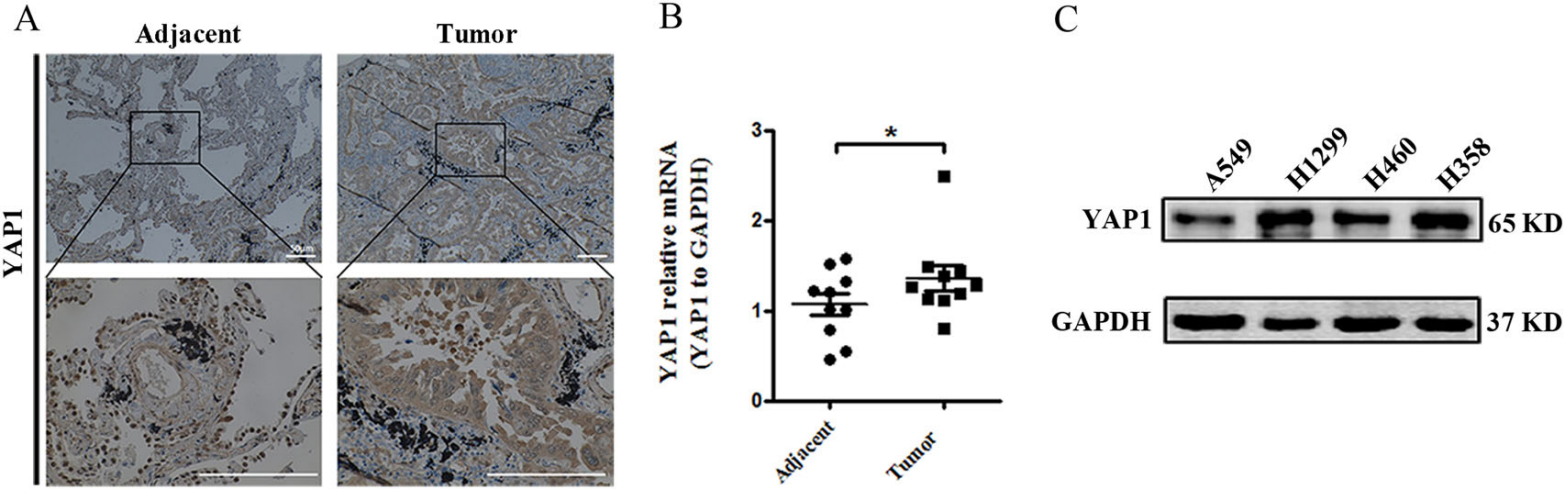
YAP1 expression levels in NSCLC tissues and NSCLC cell lines. **a** Representative images of immunohistochemical (IHC) staining of YAP1 in human NSCLC tissues and matched adjacent tissues; significantly increased YAP1 staining is shown in human NSCLC tissues. The scale bars indicate 50 µm. **b** Quantitative real-time RT-PCR analysis of YAP1 mRNA levels normalized to GAPDH in human NSCLC tissues and matched adjacent tissues. *n* = 10, *P < 0.05 vs. Adjacent tissues. **c** Western blotting analysis of YAP1 protein levels in the four NSCLC cell lines was performed. GAPDH was used as an internal control. *n* = 3

To determine the role of YAP1 in the development of NSCLC, we first examined YAP1 expression in 14 tumor samples by immunohistochemistry (IHC) assays; we found that YAP1 expression was obviously higher in NSCLC tissues than in paired adjacent tissues (Fig. 1a). Consistently, real-time RT-PCR analyses demonstrated that the mRNA expression levels of YAP1 were significantly higher in NSCLC tissues than in adjacent tissues (Fig. 1b). We further evaluated YAP1 expression in various NSCLC cell lines (A549, H460, H358, and H1299). The data from western blots also showed that the protein expression levels of YAP1 were higher in NSCLC cell lines, including H1299, H358, H460, and A549 (Fig. 1c). Collectively, these results indicated the potential role of YAP1 in NSCLC progression.

### YAP1 functionally promotes NSCLC cell proliferation, migration, and invasion

**Fig. 2:**
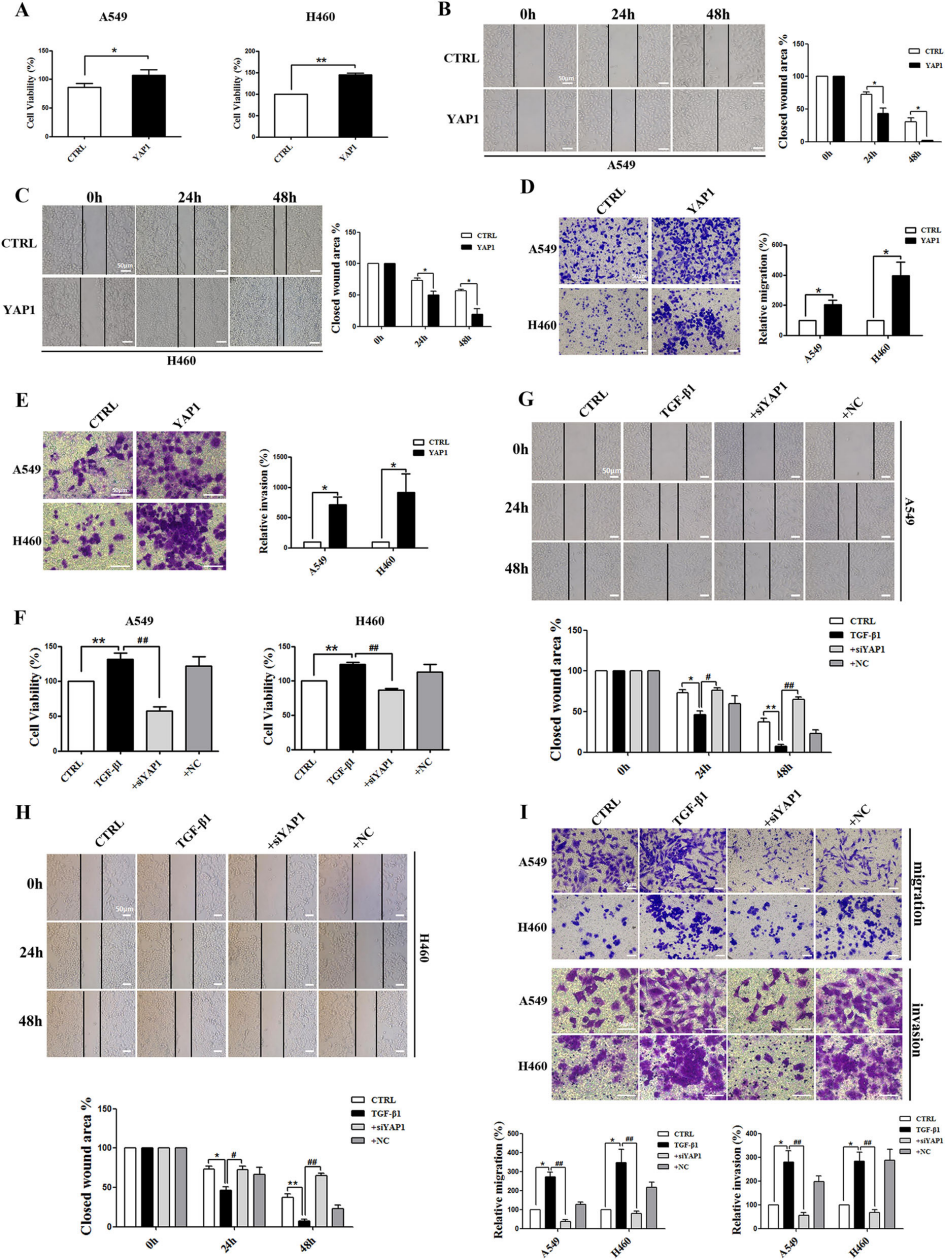
Effects of YAP1 on cell proliferation, migration, and invasion in vitro. **a** MTT analysis of cell viability in A549 and H460 cells overexpressing YAP1. Representative images from wound-healing assays using A549 cells (**b**) and H460 cells (**c**) overexpressing YAP1 at 0, 24, and 48 h after scratching (left panels). The wound-healing assay results are quantified in the histogram (right panel). Representative images of the migration (**d**) and invasion (**e**) of A549 and H460 cells overexpressing YAP1 from transwell assays (left panel). Cell counts are for the corresponding assays of at least four random microscope fields (migration: ×100 magnification; invasion: ×200 magnification). Cell migration and invasion are expressed as a percentage of the control (right panel). **f** MTT analysis of cell viability in A549 and H460 cells with YAP1 silencing. **g**–**h** Representative images from wound-healing assays using A549 and H460 cells with YAP1 silencing at 0, 24, and 48 h after scratching. The wound-healing assay results for A549 and H460 cells with YAP1 silencing are quantified in the histogram. **i** Representative images of the migration (top panel) and invasion (medium panel) of A549 and H460 cells with YAP1 silencing from transwell assays. Cell counts are for the corresponding assays of at least four random microscope fields (migration: ×100 magnification; invasion: ×200 magnification). Cell migration and invasion are expressed as a percentage of the control (bottom panel). The scale bars indicate 50 µm. The experiments were performed at least three times, and the data are presented as the mean ± SEM. *n* = 4–8; *P < 0.05, **P *<* 0.01 vs. CTRL; ^**#**^*P* < 0.05, ^**##**^*P* < 0.01 vs. TGF-β1

To identify the potential regulatory effects of YAP1 on NSCLC progression, we generated a YAP1 construct that was able to overexpress YAP1 in A549 and H460 cells. We found that YAP1 overexpression enhanced cell viability in both A549 and H460 cells (Fig. 2a). Then, we tested the autonomous migration ability of the cells by using wound-healing assays. As shown in Fig. 2b, c, the forced expression of YAP1 significantly accelerated the speed of wound closure in both A549 and H460 cells. Consistently, transwell assays further confirmed that YAP1 overexpression promoted migration and invasion in both A549 and H460 cells (Fig. 2d, e).

Next, we introduced an siRNA construct for YAP1 to further explore the role of YAP1 in NSCLC. We found that knocking down YAP1 alleviated TGF-β1-induced cell proliferation, migration, and invasion in both A549 and H460 cells (Fig. 2f–i). These results indicated that knocking down YAP1 inhibited NSCLC proliferation and motility and may be a novel target for the treatment of NSCLC.

### YAP1 regulates cell migration and invasion in NSCLC by inducing the EMT program

**Fig. 3:**
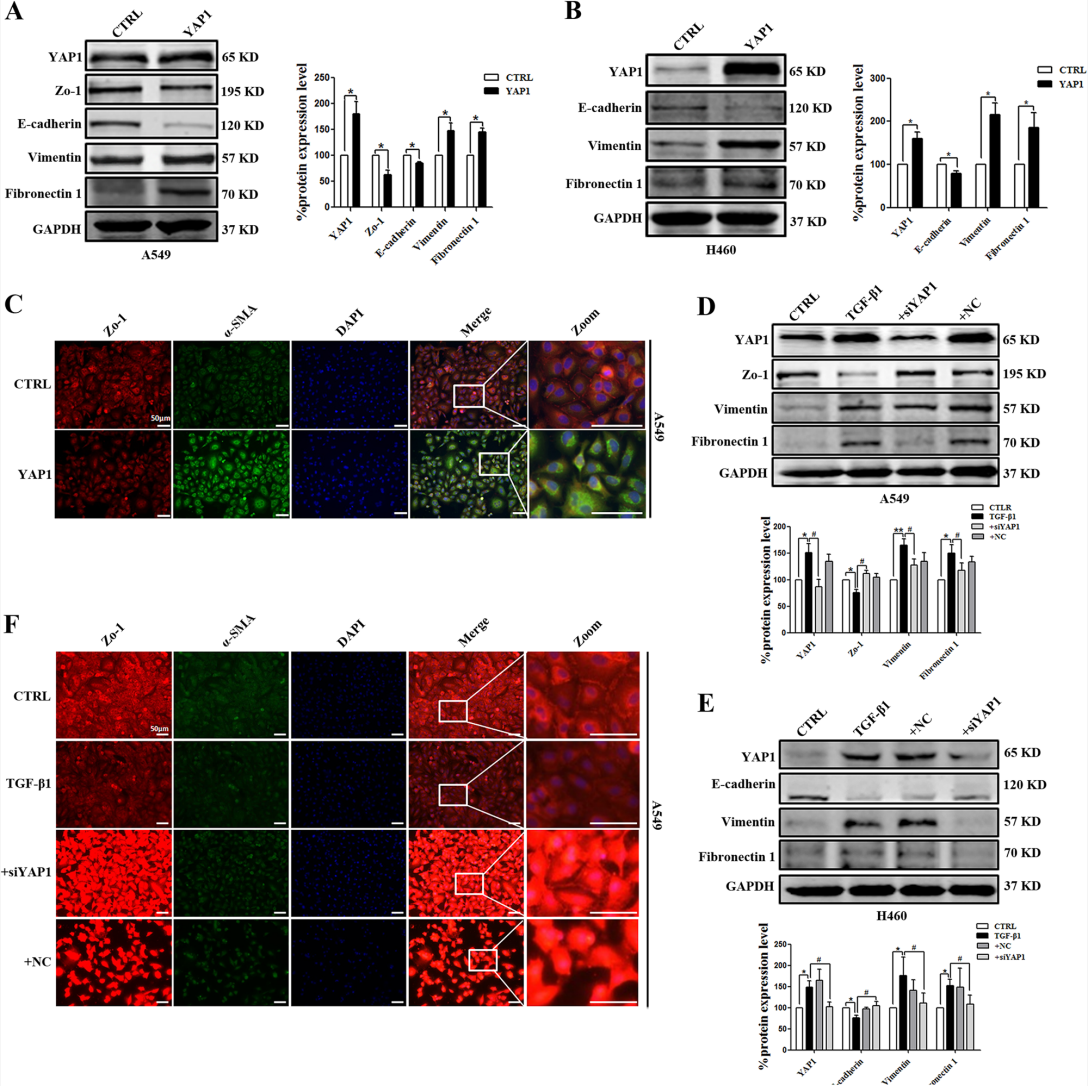
EMT-related marker expression in A549 cells with YAP1 overexpression or silencing. Western blot (**a**, **b**) and immunofluorescence assays (**c**) show that the overexpression of YAP1 promotes EMT in A549 and H460 cells. Western blot (**d**, **e**) and immunofluorescence assays (**e**) show that silencing YAP1 inhibits EMT in A549 and H460 cells. E-cadherin and Zo-1, epithelial markers. Vimentin, fibronectin 1, Slug, α-SMA, mesenchymal markers. GAPDH was used as an internal control. Zo-1 and α-SMA protein expression levels were determined by immunofluorescence in A549 cells. Zo-1 is stained red, α-SMA is stained green, and the nuclei are stained blue. The scale bars indicate 50 µm, *n* = 4; *P < 0.05, **P *<* 0.01 vs. CTRL; ^**#**^*P* < 0.05, ^**##**^*P* < 0.01 vs. TGF-β1

EMT is considered to be a pivotal step for tumor infiltration and distant metastasis in a variety of carcinomas. Thus, we hypothesized that YAP1 contributed to NSCLC by disturbing the EMT program. To investigate this hypothesis, we first determined whether the expression levels of epithelial markers (E-cadherin and Zo-1) and mesenchymal markers (vimentin and fibronectin 1) changed under conditions of abnormal YAP1 expression. As shown in Fig. 3a, b, YAP1 overexpression resulted in the downregulation of E-cadherin and Zo-1 and the upregulation of vimentin and fibronectin 1 in A549 and H460 cells. In addition, immunofluorescence assays further showed a reduction in the intensity of Zo-1 staining, and α-SMA displayed peak staining in the YAP1 overexpression group (Fig. 3c). Furthermore, TGF-β1 had the same effects on YAP1 overexpression, but YAP1 inhibition abolished these effects (Fig. 3d, f). Taken together, these data suggested that YAP1 contributed to NSCLC migration and invasion by inducing the EMT program.

### TEAD is involved in EMT in YAP1-induced NSCLC

**Fig. 4:**
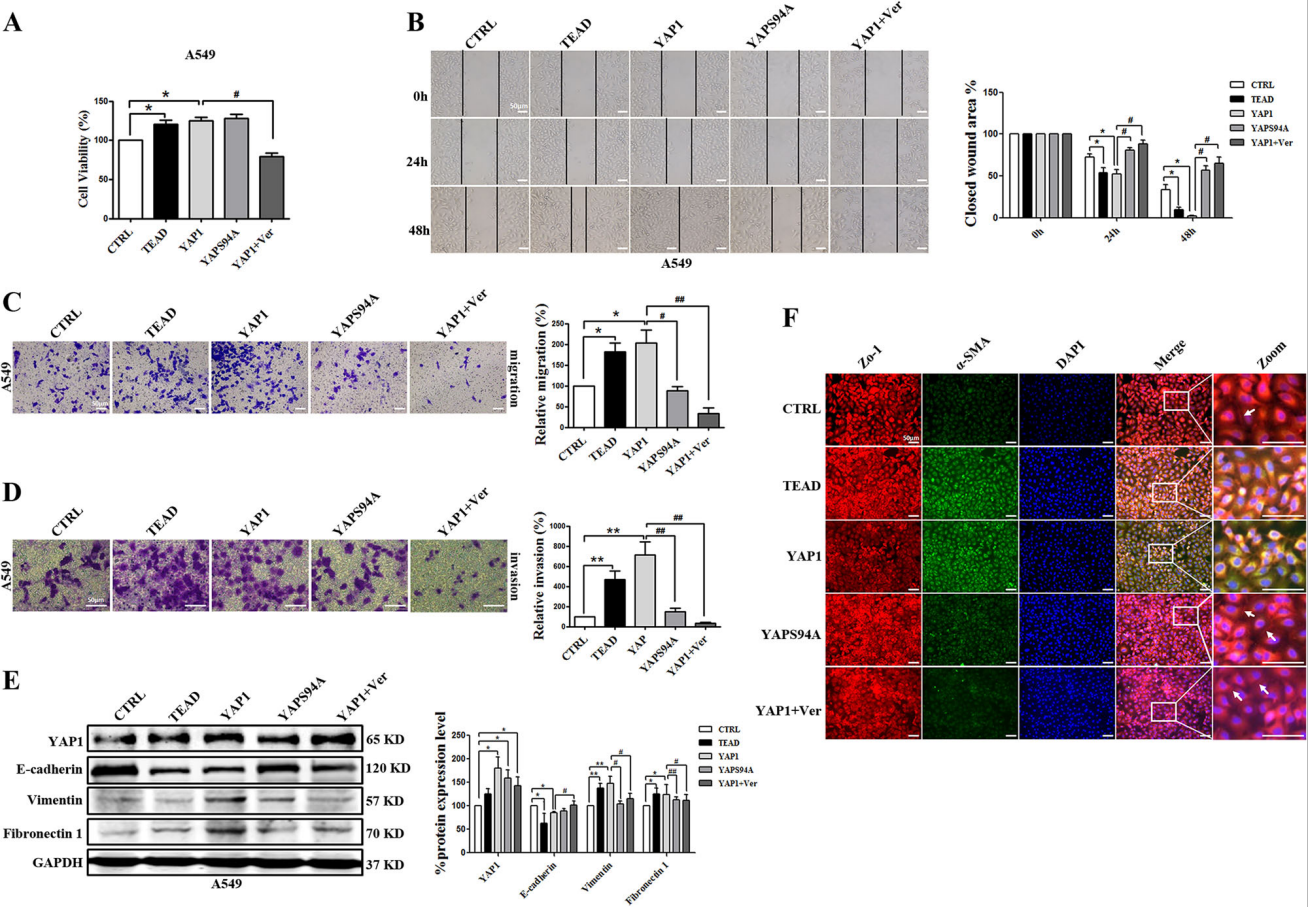
TEAD is involved in YAP1-induced EMT in A549 cells. **a** MTT analysis of cell viability in A549 cells shows that verteporfin inhibits cell proliferation. **b** Representative images from wound-healing assays using A549 cells at 0, 24, and 48 h after scratching show that compared with YAP1 overexpression, verteporfin inhibits cell migration, and YAPS94A has no effect on cell migration (left panels). The wound-healing assay results are quantified in the histogram (right panel). Representative images of cell migration (**c**) and invasion (**d**) show that compared with YAP1 overexpression, verteporfin inhibits A549 cell migration and invasion, and YAPS94A has no effect on A549 cell migration and invasion (left panel). Cells counts are for the corresponding assays of at least four random microscope fields (migration: ×100 magnification; invasion: ×200 magnification). Cell migration and invasion are expressed as a percentage of the control (right panel). **e** The western blots show that EMT-related markers are differentially expressed in the verteporfin group compared with the YAP1 overexpression group, and YAPS94A has no effect on EMT-related markers expression in A549 cells. GAPDH was used as an internal control. **f** Immunofluorescence assays show that verteporfin upregulates the staining intensity of Zo-1 and downregulates the staining intensity of α-SMA in A549 cells, but YAPS94A has no effect on the staining intensities of Zo-1 and α-SMA. Zo-1 is stained red, α-SMA is stained green, and the nuclei are stained blue. The scale bars indicate 50 µm. The experiments were performed at least three times, and the data are presented as the mean ± SEM. *n* = 4–8; *P < 0.05, **P < 0.01 vs. CTRL; ^**#**^*P* < 0.05, ^**##**^*P* < 0.01 vs. YAP1

TEAD, a co-transcriptional activator of YAP1, mediates YAP-induced cell growth, oncogenic transformation, and EMT in breast cancer^33,34^. To confirm whether TEAD plays essential roles in mediating the biological function of YAP1 in NSCLC, YAPS94A (a YAP1 mutant missing the TEAD binding site), and verteporfin were used to disrupt the interaction between YAP1 and TEAD^33,35^. As illustrated in Fig. 4a–d, the overexpression of TEAD or YAP1 promoted A549 cell proliferation, migration, and invasion, whereas YAPS94A failed to promote migration and invasion, even though it promoted A549 cell proliferation. Moreover, verteporfin pre-treatment of A549 cells attenuated the effects of YAP1 on those capabilities (Fig. 4a–d). Consistent with these results, YAPS94A had no effects on migration and invasion, but it promoted cell proliferation; verteporfin mitigated the effects of YAP1 on cell migration and invasion in H460 cells (Supplement Figs. 1A-1D). In addition, compared with YAP1 overexpression in A549 and H460 cells, verteporfin pre-treatment alleviated YAP1-induced EMT, which was indicated by the upregulation of E-cadherin and Zo-1 and the downregulation of the mesenchymal markers vimentin and fibronectin 1(Fig. 4e, f and Supplement Fig. 1E). Furthermore, YAPS94A had no significant effects on EMT-related markers (Fig. 4e, f and Supplement Fig. 1E). Our data suggested that TEAD was a necessary mediator of YAP1-induced EMT in NSCLC.

**Fig. 5:**
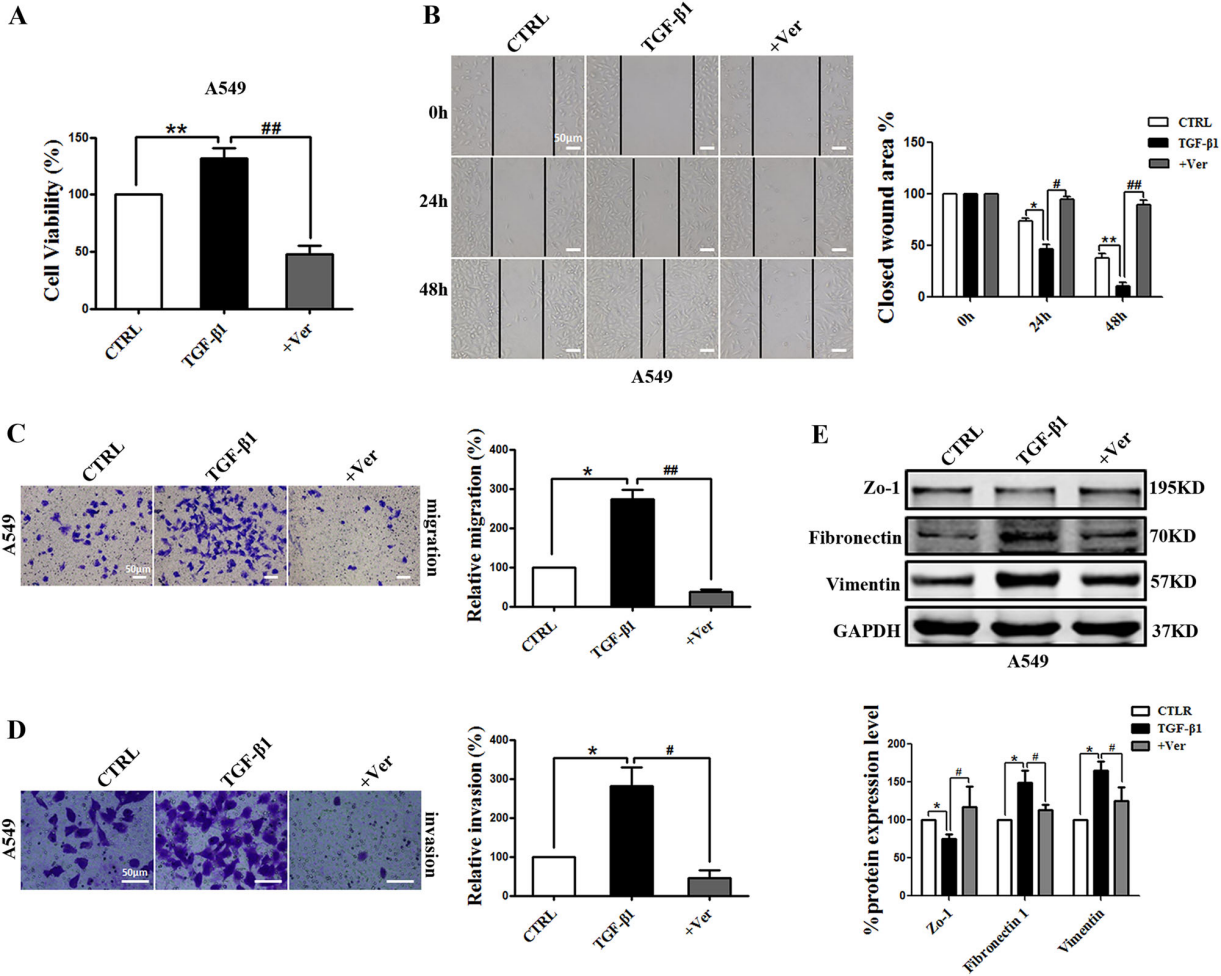
Inhibiting the co-transcription complex YAP/TEAD reverses A549 cell proliferation, migration, and invasion induced by TGF-β1. **a** MTT analysis of cell viability in A549 cells shows that verteporfin inhibits cell proliferation induction by TGF-β1. **b** Representative images from wound-healing assays using A549 cells at 0, 24, and 48 h after scratching show that verteporfin inhibits cell migration induction by TGF-β1 (left panels). The wound-healing assay results are quantified in the histogram (right panel). Representative images of the migration (**c**) and invasion (**d**) of A549 cells show that verteporfin inhibits cell migration and invasion induction by TGF-β1 (left panel). Cells counts are for the corresponding assays of at least four random microscope fields (migration: ×100 magnification; invasion: ×200 magnification). Cell migration and invasion are expressed as a percentage of the control (right panel). **e** The western blots show that verteporfin reverses EMT-related markers expression induction by TGF-β1 in A549 cells. GAPDH was used as an internal control. The scale bars indicate 50 µm. The experiments were performed three times, and the data are presented as the mean ± SEM. *n* = 4–8; *P < 0.05, **P < 0.01 vs. CTRL; ^**#**^*P* < 0.05, ^**##**^*P* < 0.01 vs. TGF-β1

Inhibition of the interaction between YAP1 and TEAD was used to further confirm the important role of TEAD. Verteporfin inhibited cell proliferation, migration, and invasion in A549 (Fig. 5a–d) and H460 cells (Supplement Figs. 2A-2D); these effects were driven by TGF-β1. In addition, verteporfin recovered the expression levels of the mesenchymal markers vimentin and fibronectin 1 and the epithelial marker E-cadherin in A549 (Fig. 5e) and H460 cells (Supplement Fig. 2E).

### Slug is a direct target of YAP1/TEAD

**Fig. 6:**
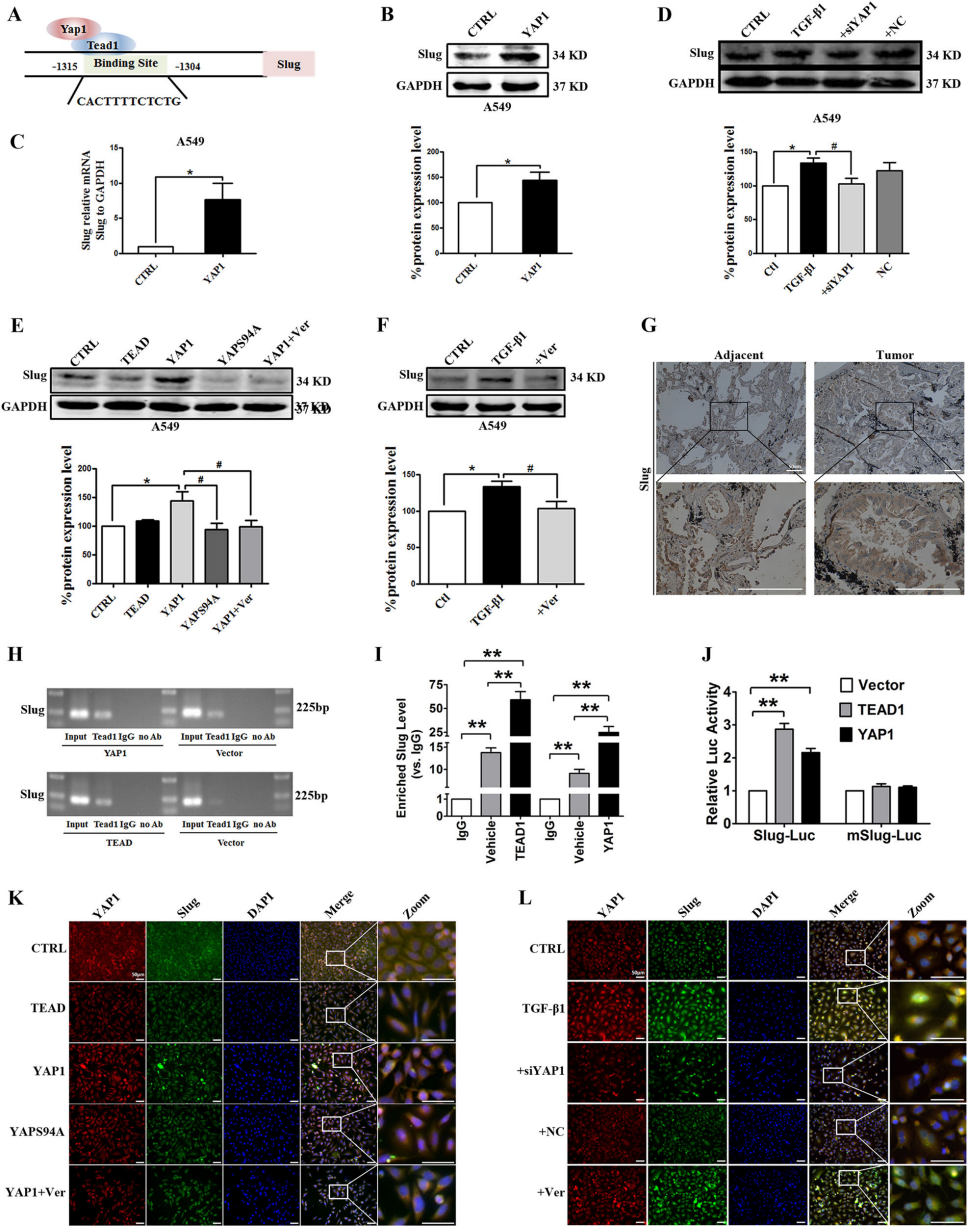
Slug is regulated by the co-transcriptional complex YAP1/TEAD in the EMT program of A549 cells. **a** The gene sequence analysis shows a putative TEAD binding site in the promoter of Slug. Western blot (**b**) and real-time RT-PCR (**c**) assays show that YAP1 overexpression upregulates the protein and mRNA levels of Slug in A549 cells. GAPDH was used as an internal control. Slug mRNA levels were normalized to GAPDH. *P < 0.05 vs. CTRL. **d**–**f**. Western blotting was used to analyze the expression of Slug in A549 cells. GAPDH was used as an internal control. **d** YAP1 silencing decreases Slug protein levels in A549 cells. *P < 0.05 vs. CTRL; ^**#**^*P* < 0.05 vs. TGF-β1. **e** Verteporfin inhibits Slug expression, and YAPS94A has no effect on Slug expression in A549 cells. *P < 0.05 vs. CTRL; ^**#**^*P* < 0.05 vs. YAP1. **f** Verteporfin reverses the upregulation of Slug via TGF-β1 in A549 cells. *P < 0.05 vs. CTRL; ^**#**^*P* < 0.05 vs. TGF-β1. **g** Representative images of the immunohistochemical (IHC) staining of Slug in human NSCLC tissues and matched adjacent tissues show that a significant increase in YAP1 staining is found in human NSCLC tissues. *n* = 10. qPCR (**h**) and chromatin immunoprecipitation (ChIP) (**i**) assays demonstrate the physical interaction between TEAD and the promoter region of Slug. *P < 0.05 vs. IgG. **j** Luciferase assays confirm that TEAD can active the transcription of Slug-luc but not mSlug-luc. *P < 0.05 vs. Vector. **k**,** l** Immunofluorescence assays show the staining intensities of YAP1 and Slug in A549 cells. YAP1 is stained red, Slug is stained green, and the nuclei are stained blue. The scale bars indicate 50 µm. The experiments were performed three times, and the data are presented as the mean ± SEM. *n* = 4–8

By using gene sequence analysis, we found a putative binding site of the co-transcriptional activators YAP1/TEAD in the promoter of Slug (Fig. 6a). In addition, we found that YAP1 overexpression upregulated Slug expression at the protein and mRNA levels (Fig. 6b, c). Nevertheless, the deletion of YAP1 or the inhibition of the YAP1/TEAD interaction by verteporfin reduced Slug expression levels in A549 (Fig. 6d–f) and H460 cells (Supplement Figs. 3A-3D). Moreover, YAPS94A had no significant effect on Slug expression under any condition in A549 (Fig. 6e) and H460 cells (Supplement Fig. 3C). In particular, Slug expression levels were much higher in tumor tissues than in adjacent tissues **(**Fig. 6g**)**; YAP1 was also highly expressed in tumor tissues. Thus, these results suggested that the ability of YAP1 to promote EMT likely involved the activation of Slug expression.

Slug can suppress E-cadherin expression by directly binding to the E-cadherin promoter; thus, it is mediator of the EMT program in many epithelial tumors, such as in lung cancer progression induced by N-α-acetyltransferase D^36,37^. Thus, we tested the possibility that Slug is a mediator of EMT in YAP1-induced NSCLC and that Slug is the target gene of the co-transcriptional activators YAP1/TEAD. Chromatin immunoprecipitation (ChIP) assays revealed that compared with vehicle treatment, YAP1 or TEAD overexpression could enrich the promoter regions of Slug (Fig. 6h), and qPCR assays demonstrated the physical interaction between TEAD and the promoter region of Slug; furthermore, YAP1 overexpression promoted the interaction between TEAD and the Slug promoter **(**Fig. 6i**)**. Consistent with these results, luciferase assays showed that overexpressing YAP1 promoted the luciferase activity of the Slug-Luc promoter, whereas overexpressing YAP1 had no influence on the luciferase activity of the mSlug-Luc promoter (Fig. 6j); these results indicated that YAP1/TEAD positively regulated Slug expression via transcriptional activation. These results illustrate that Slug is the target gene of the co-transcriptional activators YAP1/TEAD.

Consistently, the immunofluorescence staining results showed that overexpressing YAP1 promoted the expression of Slug in the nucleus, but cells overexpressing YAPS94A and treated with verteporfin had low expression levels of Slug (Fig. 6k). In addition, silencing YAP1 or adding verteporfin also decreased Slug expression levels compared with those in the TGF-β1 group (Fig. 6l). These data suggested that the YAP1/Slug correlation was associated with YAP1/TEAD-dependent transcriptional activity but was not correlated with TEAD-dependent transcriptional activity. The data also provides further evidence that Slug is capable of mediating YAP1 activity in EMT and thus contributes to NSCLC.

### YAP1 knockdown inhibits tumorigenesis and EMT in vivo

**Fig. 7:**
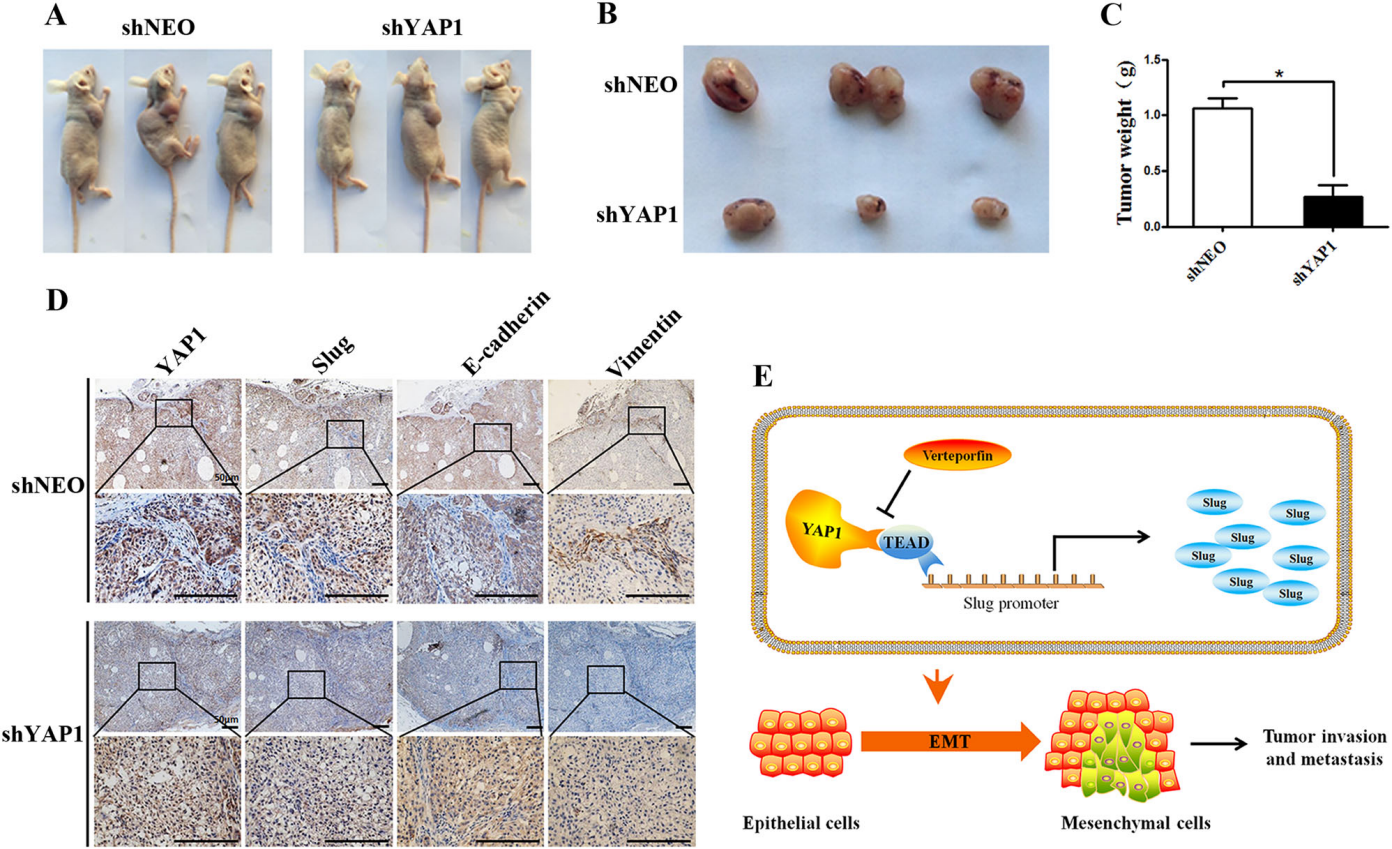
YAP1 regulates tumorigenesis and EMT in vivo. **a** Representative images of tumorigenesis after the subcutaneous injection of luc-shRNA-hYAP1-NEO or luc-shRNA-NEO cells into a xenograft nude mouse model. shYAP1: luc-shRNA-hYAP1-NEO cells; shNEO: luc-shRNA-NEO cells. **b** Effects of YAP1 knockdown on the size of A549 xenograft tumors in nude mice. **c** The significantly smaller average tumor weights of the shYAP1 group are compared to their counterparts in the shNEO group. **d** Representative images of the IHC staining of YAP1, Slug, E-cadherin, and vimentin in the xenograft tumors from nude mice. The scale bars indicate 50 µm. *n* = 3; *P < 0.01. **e** Model of the role of YAP1 in NSCLC

Next, to verify the effects of YAP1 on NSCLC tumorigenesis and the EMT program in an in vivo model, we generated a luciferase-labeled stable YAP1 knockdown human lung cancer A549 cell line (Luc-shRNA-hYAP1-NEO) and a scrambled shRNA human lung cancer A549 cell line (Luc-shRNA-Scramble-NEO). Then, these cells were subcutaneously injected into nude mice. After 4 weeks, the mice receiving YAP1 knockdown cells exhibited significant NSCLC growth compared with the mice receiving scrambled shRNA cells (Fig. 7a, b). Moreover, compared with scrambled shRNA, YAP1 knockdown significantly decreased the tumor weights (Fig. 7c). Furthermore, IHC analyses revealed that tumors from the YAP1 knockdown group exhibited less YAP1 and Slug staining, which indicated that YAP1 suppressed the expression of Slug in vivo. In addition, YAP1 knockdown resulted in a prominent increase in E-cadherin staining, as well as less vimentin staining compared to that in tumors formed by the control cells. These data indicated that nude mice injected with YAP1 knockdown cells exhibited only a small amount of tumorigenesis (Fig. 7d).

## Discussion

Up to now, Hippo signaling has been proposed to be associated with the tumorigenicity of many tumors. YAP1, a regulator of cell fate, is upregulated in multiple cancers and is significantly associated with histological differentiation, TNM stage, and poor prognosis in cholangiocarcinoma (CCA) and colorectal cancer^38–41^. In this study, we show that TEAD-mediated YAP1 promotes the transcription of Slug to induce NSCLC migration and invasion. This process is depicted in the model shown in Fig. 7e. In vivo and in vitro assays confirmed that increased expression levels of YAP1 promoted cell proliferation, migration, and invasion, whereas silencing YAP1 significantly inhibited migration, invasion, and cell growth, suggesting that YAP1 is a key regulator of cell migration, invasion, and tumorigenesis in NSCLC progression.

More and more studies suggest that EMT is a pivotal step required for epithelial cells to acquire malignant capabilities^5,7^. YAP1 has been shown to be closely linked to the EMT program in CCA and breast cancer^34^. We also found that YAP1 could induce EMT in NSCLC. As expected, YAP1 overexpression resulted in a decrease in epithelial markers and an increase in mesenchymal markers, whereas silencing YAP1 had opposite effects. Slug, a key EMT regulator, is best known for its role in orchestrating EMT programs associated with development^42–44^. Recently, Yi Tang et al. suggested that SNAI1 and Slug impacted stem cell functions and bone formation via cooperative interaction with YAP/TAZ^45,46^, but whether there is a direct interaction between Slug and YAP1 that could induce EMT has not been described previously. In our study, we found that YAP1 induced the EMT program in NSCLC through regulating the transcription of Slug by interacting with TEAD. These data suggest that this transcriptional regulation between YAP1/TEAD and Slug may impact stem cell functions and bone formation; these actions deserve further investigation in the future.

In addition, YAP1 is a TF that lacks a DNA-binding motif^24,47^. A large body of evidence found that members of the TEAD family of TFs were critical partners of YAP1 in regulating gene expression^24,33^. In our study, we also demonstrate that TEAD plays a major role in mediating Slug expression in NSCLC. However, a YAP1 mutant missing the TEAD binding site, YAPS94A, could still promote cell proliferation, suggesting that YAP1 regulates NSCLC proliferation by regulating other target genes that are mediated by other TFs. Based on these results, we believe that Slug participates in NSCLC progression. However, given the capacity of YAP1 to regulate the expression of multiple genes that are involved in many biological processes, we cannot exclude that other genes that are directly regulated by YAP1 contribute to NSCLC invasion and migration.

Identification of a reliable biomarker for prognosis and related mechanisms in NSCLC will provide new options for diagnosis. Our current study supports that YAP1 and Slug levels are significantly higher in patient tumor specimens than in adjacent tissues. Furthermore, YAP1 and Slug have important roles in promoting cancer cell invasion and migration in vitro, and silencing YAP1 inhibits NSCLC formation and EMT in vivo. We propose that YAP1 and Slug might be useful markers of NSCLC formation, but this finding requires further investigation.

In summary, we have demonstrated for the first time that YAP1 promotes NSCLC tumorigenesis and metastasis by regulating the transcription of Slug in a YAP1/TEAD-dependent manner. Our study reveals a previously unrecognized pathway and explains a new mechanism of YAP1 and EMT in NSCLC; these results thus suggest several novel therapeutic targets, including YAP1 and Slug.

## Materials and methods

### Lung tissues

Fresh lung cancer tissue samples and adjacent normal tissue samples were taken from lung adenocarcinoma patients undergoing surgical procedures at the Second Affiliated Hospital of the Harbin Medical University (Harbin, China). All of the patients or their guardians provided written consent, and the Ethics Committee of Harbin Medical University approved all aspects of this study.

### IHC assay

Human tissues slides were obtained from the Second Affiliated Hospital of the Harbin Medical University. Briefly, immunostaining was performed on 5-μm-thick tissue sections. The sections were dewaxed and deparaffinized in xylene and rehydrated in graded alcohol solutions. The antigen-retrieval process was performed by heating the sections for 30 min in Tris-EDTA buffer. The slides were subsequently stained with primary antibodies for YAP1 (Proteintech, 13584-1-AP, 1:50), Slug (Cell Signaling, #9585, 1:100), E-cadherin (Cell Signaling, #9562, 1:100), vimentin (Cell Signaling, #5741, 1: 100), and their respective secondary antibodies. The sections were then counter-stained with hematoxylin, followed by dehydration and mounting. Images were captured with an Olympus camera.

### Cell culture, reagents, and expression constructs

The lung cancer cell lines A549, H460, H358, and H1299 were purchased from the Cell Bank of the Chinese Academy of Sciences (Shanghai, China). In addition, A549-Luc-Puro-shRNA-hYAP1-Neo, A549-Luc-Puro-shRNA-Neocells were purchased from Biowit Technologies (Shenzhen, China). Cell were cultured in F12K (GIBCO, NY, USA), PRIM 1640 (GIBCO, NY, USA), or DMEM (GIBCO, NY, USA) supplemented with 10% fetal bovine serum (FBS, BI) and 1% penicillin/streptomycin at 37 °C and 5% CO_2_. The expression plasmids encoding YAP1, YAPS94A, TEAD, Slug-Luc, and mSlug-Luc were constructed using PGL3; verteporfin was purchased from Sigma.

### Wound-healing and transwell assays

For wound-healing assays, cells were seeded at a density of 1 × 10^6^ cell/well in six-well plates. An artificial wound was created on the confluent cell monolayer 6 h after transfection using a sterile 10-µl pipette tip. The suspended cells were washed away with PBS, and the cells were then cultured in medium with 2% FBS (Biological Industries, Cromwell, CT, USA). The wounds were photographed with a light microscope at 0, 24, and 48 h after treatment. In vitro cell migration and invasion were investigated using a 24-well insert transwell migration assay and a Matrigel invasion assay (8.0 µm, Corning, NY, USA). For the migration assay, 5 × 10^4^ cells were suspended in 200 µl of serum-free F12k/RPIM1640 (Gibco, Life Technologies, Carlsbad, CA, USA) and placed in the top chambers. For the invasion assay, 2 × 10^5^ cells were suspended in 200 µl of F12k/RPIM1640 without serum and then seeded on the cell culture insert pre-coated with 1 µg/µl Matrigel (BD Biosciences, USA). Complete medium was added to the bottom wells to stimulate migration or invasion. After incubation for 48 h, the cells that did not penetrate through the membrane were removed with a cotton swab, while those adhered to the lower surface of the membrane were stained with a 0.1% crystal violet solution. The number of migrated cells in five randomly selected fields was counted under a light microscope (magnification, x200; Olympus, Tokyo, Japan).

### Immunofluorescence assay

For immunofluorescence assays, cells were seeded at a density of 1 × 10^6^ cell/well on coverslips in 24-well plates. After transfection, the coverslips were fixed in cold methanol for 20 min, and the cells were washed thoroughly with PBS-Tween (PBST). Then, the cells were permeabilized with 0.1% Triton X-100 for 1 h and washed thoroughly with PBST. Lastly, the cells were blocked with goat serum for 40 min at room temperature, followed by incubation with primary antibodies overnight. The cells were washed five times with PBST and then incubated with Alexa Fluor 488-tagged or Alexa Fluor 594-tagged secondary antibodies (Life Technologies). After washing with PBST, the nuclei were counterstained with 4,6-diamidino-2-phenylindole (DAPI, Beyotime Biotechnology, China). Imaging was performed under a Zeiss fluorescence microscope equipped with an epifluorescence and Axiocam camera system and Axiovision software (Carl Zeiss, Oberkochen, Germany).

### Tumorigenesis in nude mice

Male BALB/c nude mice (5 weeks old) were purchased from Beijing Vital River Laboratory Animal Technology (Beijing, China) and maintained in pathogen-free conditions. For tumor growth, nude mice were injected subcutaneously with 1 × 10^6^ NSCLC cells, including A549-Luc-Puro-shRNA-hYAP1-Neo, A549-Luc-Puro-shRNA-Neo (*n* = 6 mice per group). Mice were sacrificed after 4 weeks, and the tumors were removed for assessment; body weights and tumor sizes were also measured. Lung tissues were collected for standard histopathology assays. All animal experiments were carried out according to the guidelines of the Ethical Committee of Harbin Medical University.

### Real-time RT-PCR

Total RNA was isolated from lung tissues or cultured cells using Trizol reagent (Invitrogen, Carlsbad, CA, USA) according to the manufacturer’s instructions. RNA integrity, quantity, and purity were examined using a Nano-Drop 8000 Spectrophotometer (Thermo Scientific, Wilmington, DE, USA). As delineated in our previous work^48^, cDNA was generated using a High Capacity cDNA Reverse Transcription Kit (Applied Biosystems, Foster City, CA, USA). Real-time PCR was performed on an ABI7500 FAST real-time PCR System (Applied Biosystems) for 40 cycles. After the reaction cycles, the threshold cycle (Ct) values were determined, and the relative mRNA levels were calculated based on the Ct values and normalized to the GAPDH level in each sample. Primer sets for YAP1 and Slug were purchased from Guangzhou RiboBio (Guangdong, China). The expression levels of GAPDH were used as internal controls; GAPDH was used for mRNA transcripts. Fold-changes in the expression of mRNA among the RNA samples were calculated.

### Western blotting

For western blot analyses, total protein was extracted from the cells. Approximately 40 µg of crude protein was denatured and electrophoresed on 10% SDS-PAGE gels. After electrophoretic separation, proteins were transferred onto nitrocellulose membranes (Merck Millipore, R7BA46025) by electro-blotting and then blocked for 70 min at room temperature in PBS containing 5% nonfat milk; the blots were probed with primary antibodies, and GAPDH was used as an internal control. The blots were incubated with YAP1 (Proteintech, 13584-1-AP, 1:750), Slug (Cell Signaling, #9585, 1:300), E-cadherin (Cell Signaling, #9562, 1:1000), vimentin (Cell Signaling, #5741, 1: 1000), Zo-1 (Proteintech, 21773-1-AP, 1:500), and GAPDH (ABclonal, AC002, 1:1000) primary antibodies in PBS at 4 °C overnight. The membranes were washed with PBS-T and then incubated with secondary antibody (Alexa Fluor) for 1 h at room temperature. Finally, images of the western blot bands were collected with an imaging system (Odyssey, LICOR, USA) and quantified by measuring the intensity in each group with Odyssey v1.2 software; GAPDH was used as an internal control. The results are expressed as fold-changes, and the data are normalized to the control values.

### Statistical analysis

All data analyses in this study were carried out using GraphPad Prism 7 (GraphPad Software) for Mac OS. Quantifications were performed using at least three independent experimental groups. When only two groups were compared, statistical analyses between groups were performed using two-tailed Student’s *t*-tests to determine significance. *P* values of less than 0.05 were considered significant. Error bars on all graphs are presented as the SEM of the mean unless otherwise indicated.

## Electronic supplementary material

Supplemental Figures
