# Supplementary material for: YAP1 contributes to NSCLC invasion and migration by promoting Slug transcription via the transcription co-factor TEAD
Source: Cell Death Dis. 2018 Apr 27;9(5):464. doi: 10.1038/s41419-018-0515-z (PMC5920099; doi:10.1038/s41419-018-0515-z)
Supplement: Supplementary file 1 — Supplemental Figures [file 41419_2018_515_MOESM1_ESM.docx]

**
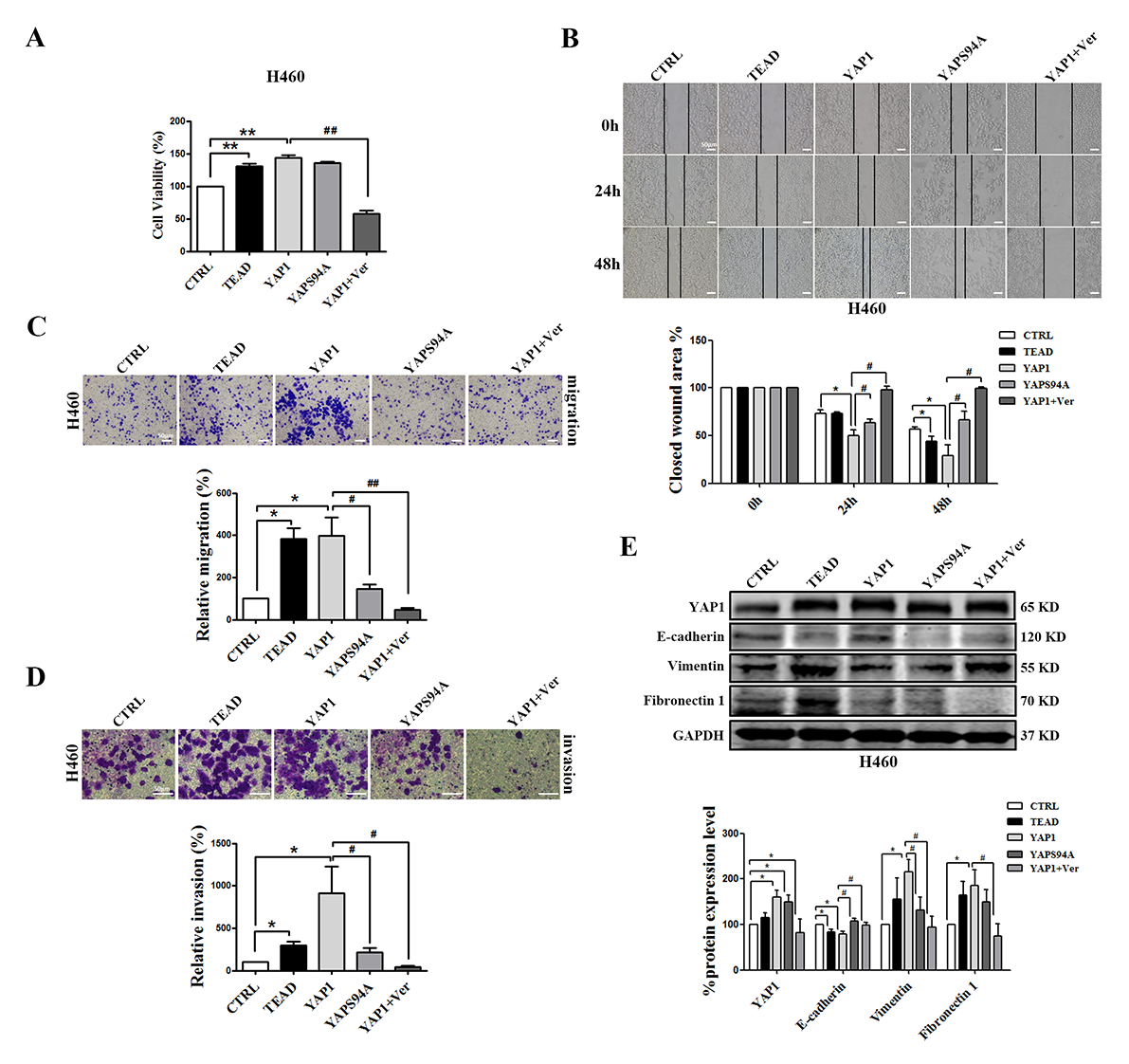
**

**Supplement figure 1. TEAD mediates YAP1-induced EMT in H460 cells.** A. MTT analysis of cell viability in H460 cells shows that verteporfin inhibits cell proliferation. **B**. Representative images from wound healing assays using H460 cells at 0, 24 and 48 h after scratching show that compared with YAP1 overexpression, verteporfin inhibits cell migration, and YAPS94A has no effect on cell migration (top panel). The wound healing assay results are quantified in the histogram (bottom panel). Representative images of the migration (**C**) and invasion (**D**) of H460 cells show that compared with YAP1 overexpression, verteporfin inhibits cell migration and invasion, and YAPS94A has no effect on cell migration and invasion (top panel). Cell counts are for the corresponding assays of at least four random microscope fields (×migration: ×100 magnification; invasion: ×200 magnification). Cell migration and invasion are expressed as a percentage of the control (bottom panel). **E.** Western blots show that EMT-related markers are differentially expressed in the verteporfin group compared with the YAP1 overexpression group, and YAPS94A has no effect on EMT-related marker expression in H460 cells. GAPDH was used as an internal control. The scale bars indicate 50 µm. The experiments were performed at least three times, and the data are presented as the mean ± SEM. n=4-8; **P* < 0.05, ***P* < 0.01 vs CTRL; ^#^*P* < 0.05, ^##^*P* < 0.01 vs YAP1.


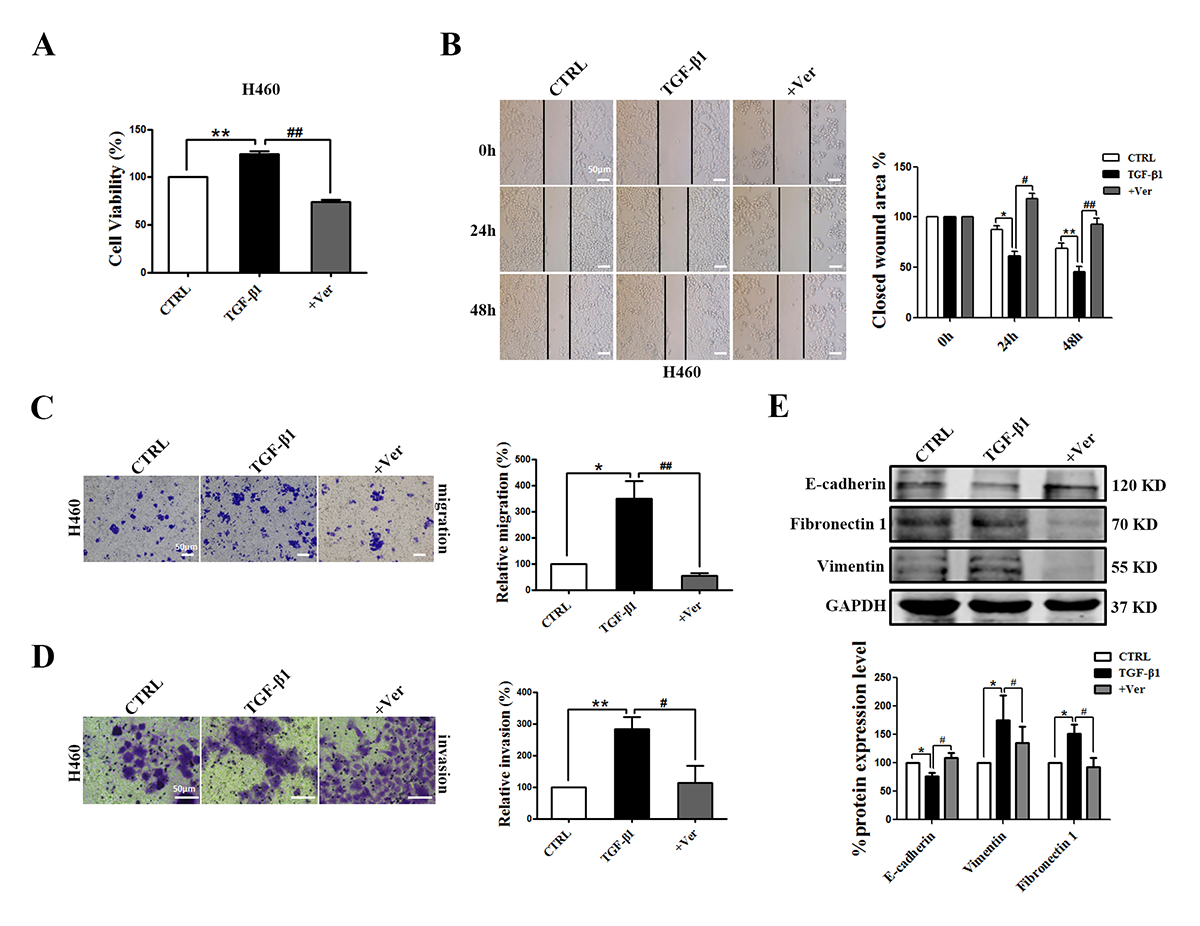


**Supplement figure 2. Inhibiting the co-transcription complex YAP/TEAD reverses H460 cell proliferation, migration and invasion induced by TGF-β1**. **A**. MTT analysis of cell viability in H460 cells shows that verteporfin inhibits cell proliferation induction by TGF-β1. **B**. Representative images from wound healing assays using H460 cells at 0, 24 and 48 h after scratching show that verteporfin inhibits cell migration induction by TGF-β1 (left panels). The wound healing assay results are quantified in the histogram (right panel). Representative images of the migration (**C**) and invasion (**D**) of H460 cells show that verteporfin inhibits cell migration and invasion induction by TGF-β1 (left panel). Cell counts are for the corresponding assays of at least four random microscope fields (migration: ×100 magnification; invasion: ×200 magnification). Cells migration and invasion are expressed as a percentage of the control (right panel). **E.** Western blots show that verteporfin reverses EMT-related marker expression induction by TGF-β1 in H460 cells. GAPDH was used as an internal control. The scale bars indicate 50 µm. The experiments were performed three times, and the data are presented as the mean ± SEM. n=4-8; **P* < 0.05, ***P* < 0.01 versus CTRL; **^#^***P* < 0.05, **^##^***P* < 0.01 vs TGF-β1.

**
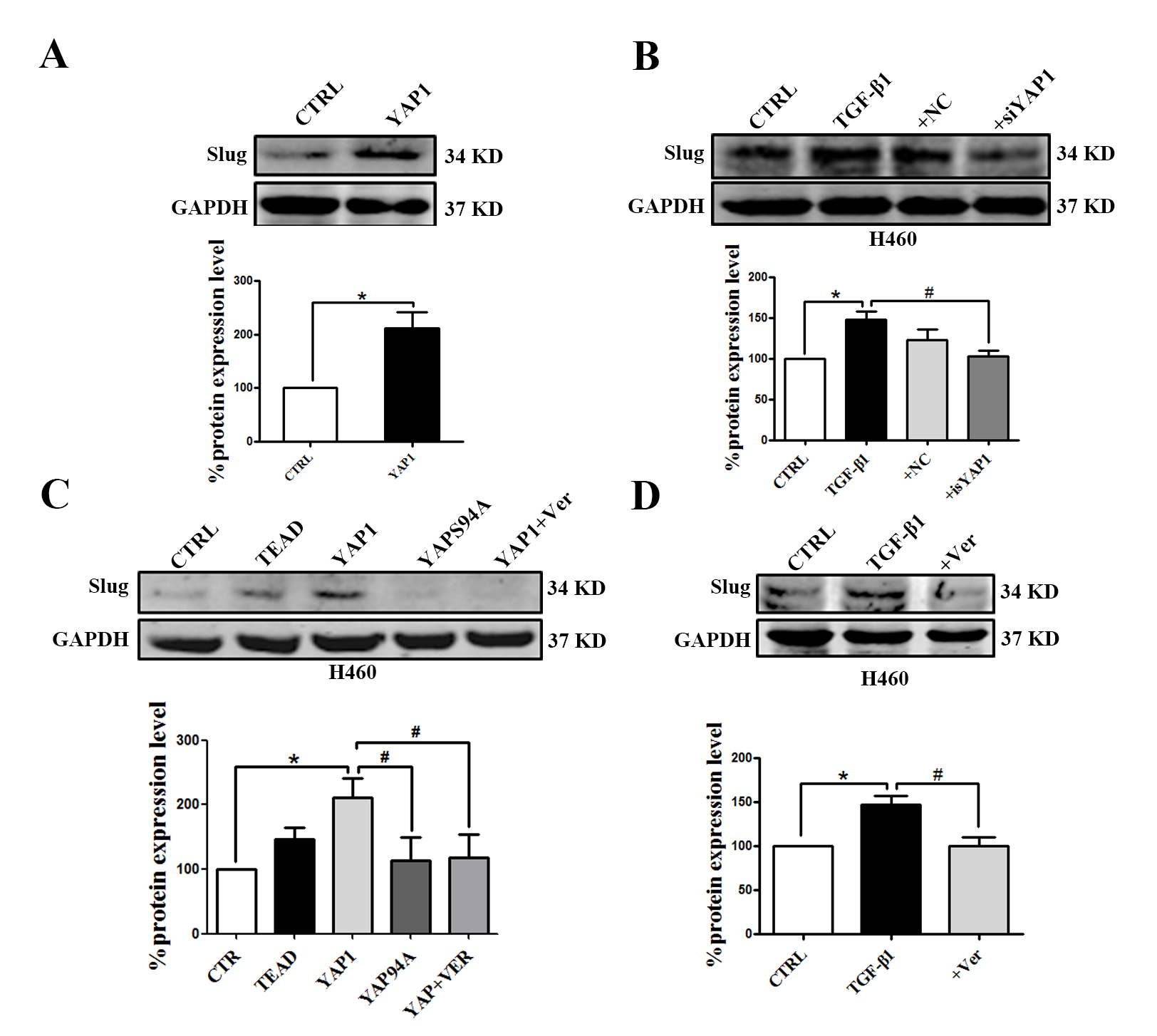
**

**Supplement figure 3. YAP1 regulates Slug expression in H460 cells. A-D.** Western blots were used to analyze the expression of Slug. **A.** YAP1 overexpression upregulates the protein levels of Slug. **P* < 0.05 vs CTRL. **B.** YAP1 silencing downregulated Slug protein levels in H460 cells. **P* < 0.05 vs CTRL; **^#^***P* < 0.05 vs TGF-β1. **C.** Verteporfin inhibits Slug expression in H460 cells. YAP-S94A has no effect on Slug expression in H460 cells. **P* < 0.05 vs CTRL; **^#^***P* < 0.05 vs YAP1. **D.** Verteporfin reverses Slug expression induced by TGF-β1 in H460 cells. The experiments were performed three times, and the data are presented as the mean ± SEM. n=4-8.
